# Supplementary figures and images for: Uncovering and characterizing splice variants associated with survival in lung cancer patients
Source: PLoS Comput Biol. 2019 Oct 25;15(10):e1007469. doi: 10.1371/journal.pcbi.1007469 (PMC6834284; doi:10.1371/journal.pcbi.1007469)

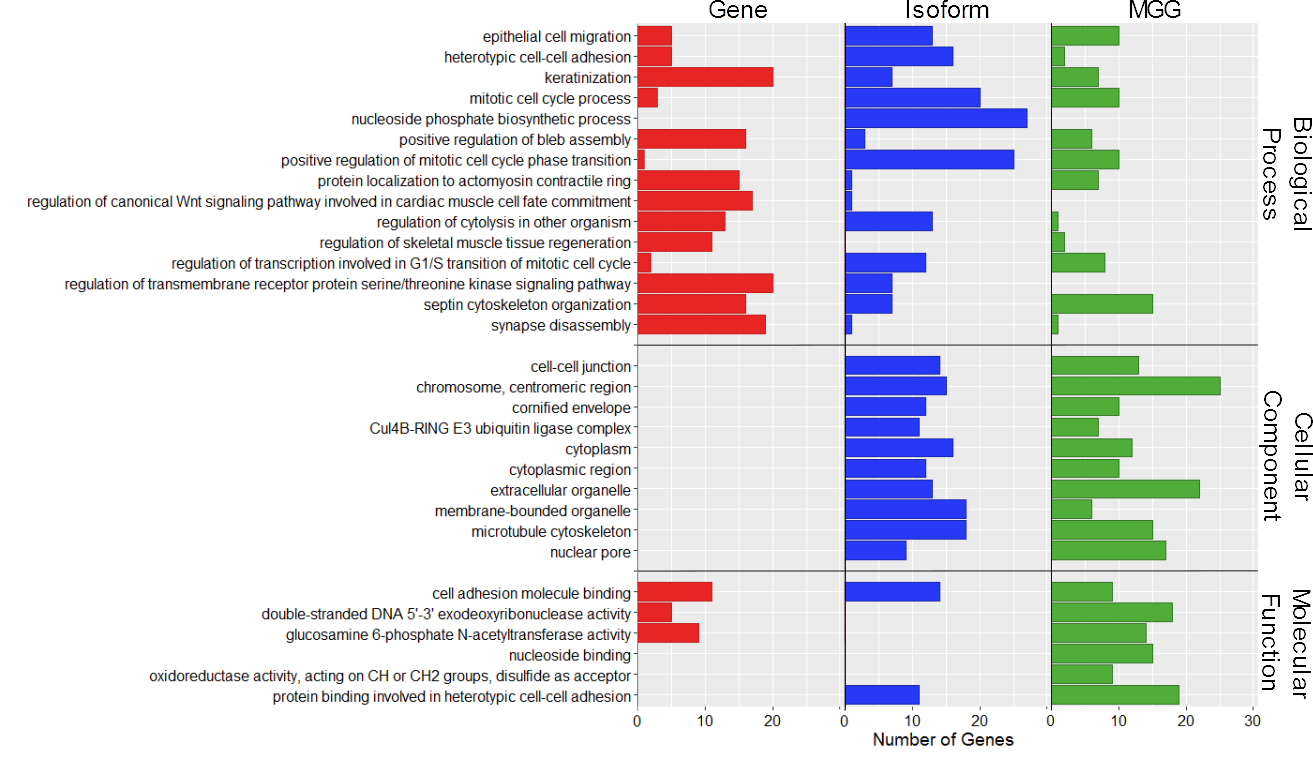

Supplement: S1 Fig — Counts of genes belonging to each enrichment cluster for the gene, isoform (splice variant), and MGG granularities are displayed as bar lengths. Missing bars does not indicate no membership, just insignificant enrichment of any term in the cluster. (TIF) [file pcbi.1007469.s001.tif]
